# A single mutation in the envelope protein modulates flavivirus antigenicity, stability, and pathogenesis

**DOI:** 10.1371/journal.ppat.1006178

**Published:** 2017-02-16

**Authors:** Leslie Goo, Laura A. VanBlargan, Kimberly A. Dowd, Michael S. Diamond, Theodore C. Pierson

**Affiliations:** 1 Viral Pathogenesis Section, National Institutes of Health, Bethesda, MD, United States of America; 2 Departments of Medicine, Molecular Microbiology, Pathology & Immunology, and The Andrew M. and Jane M. Bursky Center for Human Immunology and Immunotherapy Programs, Washington University School of Medicine, St. Louis, MO, United States of America; University of Queensland, AUSTRALIA

## Abstract

The structural flexibility or ‘breathing’ of the envelope (E) protein of flaviviruses allows virions to sample an ensemble of conformations at equilibrium. The molecular basis and functional consequences of virus conformational dynamics are poorly understood. Here, we identified a single mutation at residue 198 (T198F) of the West Nile virus (WNV) E protein domain I-II hinge that regulates virus breathing. The T198F mutation resulted in a ~70-fold increase in sensitivity to neutralization by a monoclonal antibody targeting a cryptic epitope in the fusion loop. Increased exposure of this otherwise poorly accessible fusion loop epitope was accompanied by reduced virus stability in solution at physiological temperatures. Introduction of a mutation at the analogous residue of dengue virus (DENV), but not Zika virus (ZIKV), E protein also increased accessibility of the cryptic fusion loop epitope and decreased virus stability in solution, suggesting that this residue modulates the structural ensembles sampled by distinct flaviviruses at equilibrium in a context dependent manner. Although the T198F mutation did not substantially impair WNV growth kinetics *in vitro*, studies in mice revealed attenuation of WNV T198F infection. Overall, our study provides insight into the molecular basis and the *in vitro* and *in vivo* consequences of flavivirus breathing.

## Author summary

Flaviviruses include emerging pathogens such as WNV, DENV, and ZIKV that threaten global health. Despite causing significant morbidity, effective vaccines or therapeutic agents to protect humans against many flaviviruses are lacking. Because of the importance of antibodies in flavivirus immunity and vaccine protection, much effort is focused on understanding the factors that modulate antibody recognition of flaviviruses. Virus breathing, which allows viruses to sample different conformations at equilibrium, has the potential to transiently expose otherwise inaccessible antibody epitopes. Here, we report the identification a single mutation in the envelope protein that alters the exposure of a poorly accessible epitope and the stability of both WNV and DENV through changes in the ensemble of structures sampled by the virus. For WNV, this change attenuated infection and pathogenesis in mice, suggesting that virus conformational dynamics have relevant consequences *in vivo*.

## Introduction

Flaviviruses are enveloped, positive-stranded RNA viruses typically transmitted to humans via infected ticks or mosquitoes. As many members of the flavivirus genus are emerging, they constitute a significant threat to global health. For example, approximately 390 million humans worldwide are infected annually with one of the four serotypes of dengue virus (DENV) [1]. West Nile virus (WNV) was introduced into North America in 1999 [2] and rapidly became the leading cause of arbovirus-related encephalitis in the United States [3]; Zika virus (ZIKV) emerged from Asia and Africa for the first time in 2007 and has since caused epidemics in French Polynesia [4], Oceania [5], and most recently, the Americas [6, 7]. Despite causing significant morbidity, licensed vaccines or therapeutic agents to protect humans against many flaviviruses are lacking. However, highly effective vaccines for some flaviviruses such as yellow fever virus, Japanese encephalitis virus, and tick-borne encephalitis virus are in use. The induction of a neutralizing antibody (NAb) response is a correlate of protection for these vaccines [8–12]. While a live-attenuated tetravalent DENV vaccine was recently licensed, its efficacy and durability varied by DENV serotype, pre-existing flavivirus immune status, and age of vaccine recipient [13–15]; the relationship between neutralization titer and protection for this vaccine is less clear. Because of the importance of antibodies for flavivirus immunity, a detailed understanding of flavivirus antigenic structure as well as the mechanisms of antibody-mediated neutralization is critical [16].

Assembled flavivirus particles are composed of three structural proteins: capsid (C), pre-membrane (prM), and envelope (E). The E protein, which consists of three structural domains (DI, DII, and DIII) connected to the viral membrane via a helical anchor, has critical roles in directing both the assembly of virions and their entry into cells. Flexible hinges between E protein domains enable conformational changes necessary for many steps of the viral life cycle, including fusion and maturation [17, 18]. Flaviviruses bud into the lumen of the endoplasmic reticulum as immature, non-infectious particles with a spiky surface composed of 60 icosahedrally arranged prM-E heterotrimers [19, 20]. During virus egress through the acidic environment of the trans-Golgi network, conformational changes in E expose a cleavage site within prM, which is recognized by host furin-like proteases. Cleavage of prM in the trans-Golgi network and release of the pr peptide in the extracellular environment give rise to mature and infectious virus particles covered by antiparallel E homodimers. NAbs can target epitopes in all three E protein structural domains and in quaternary structures composed of multiple domains within or across E dimers [21–31], and may block multiple steps in the virus entry pathway [32–35].

Cryo-electron microscopic (cryo-EM) reconstructions of DENV [36, 37], ZIKV [38, 39], and WNV [40] have provided detailed models for the structure of mature virions on which 90 E dimers lie flat against the viral membrane in a herringbone pattern. However, several lines of evidence suggest that many infectious flaviviruses exist as structures beyond those captured by high-resolution cryo-EM reconstructions [16]. Flaviviruses are released from infected cells as a heterogeneous population containing varying amounts of uncleaved prM due to incomplete maturation [41]. While the structures of these partially mature virions are not fully defined, they appear to contain regions that display mature- and immature-like arrangements of E proteins in varying proportions [42]. The presence of uncleaved prM on virions modulates the accessibility of many E protein epitopes recognized by NAbs [43–47]. Additionally, prM retention on virions allows for recognition by prM-reactive antibodies incapable of efficiently neutralizing infectivity. These prM-specific antibodies can enhance DENV infection and potentially contribute to severe clinical disease [48–52].

Flavivirus heterogeneity also arises from conformational flexibility of viral proteins that allows virions to sample an ensemble of conformations at equilibrium [53]. As with changes in virus maturation efficiency, virus conformational dynamics or ‘breathing’ has the potential to modulate antibody recognition and potency. Prolonged virus-antibody incubation reveals time-and temperature-dependent changes in antibody potency, the degree of which correlates generally with predictions of epitope accessibility on the mature virion; the neutralization potency of antibodies targeting cryptic epitopes can be enhanced more significantly compared to antibodies targeting highly accessible epitopes [54]. In this context, prolonged incubation of WNV and DENV in the absence of antibody does not irreversibly render them more sensitive to neutralization, suggesting that antibody binding may stabilize transiently sampled virion conformations [47].

The role of virus breathing in modulating antibody recognition is exemplified by the DENV1-specific human monoclonal antibody (mAb) E111, which neutralizes two DENV1 strains, Western Pacific (WP) and 16007, with a ~200-fold difference in potency [55]. Structural analyses revealed that E111 binds to an epitope in DIII that is predicted to be inaccessible based on existing cryo-EM models. The strain-dependent neutralization potency of E111 was neither explained by antibody binding affinity nor sequence variation within the epitope. Instead, a single residue in DII outside of the antibody footprint that differed between DENV1 WP and 16007 was responsible for modulating sensitivity to neutralization by E111 [56]. Thus, natural variation at this residue regulates the conformational dynamics of DENV1 in a way that affects exposure of the distal E111 epitope.

The determinants and functional consequences of a dynamic virion are poorly understood. In this study, we describe a single residue within the E protein DI-DII hinge that alters the neutralization sensitivity and stability of WNV and DENV virions through changes in conformational dynamics. Mutation at this residue in the WNV E protein attenuated infection and pathogenesis in mice, suggesting that changes in virus breathing have relevant consequences *in vivo*.

## Results

### Mutation of residue 198 in the WNV E DI-DII hinge increases the accessibility of a cryptic DII fusion loop epitope

**Fig 1:**
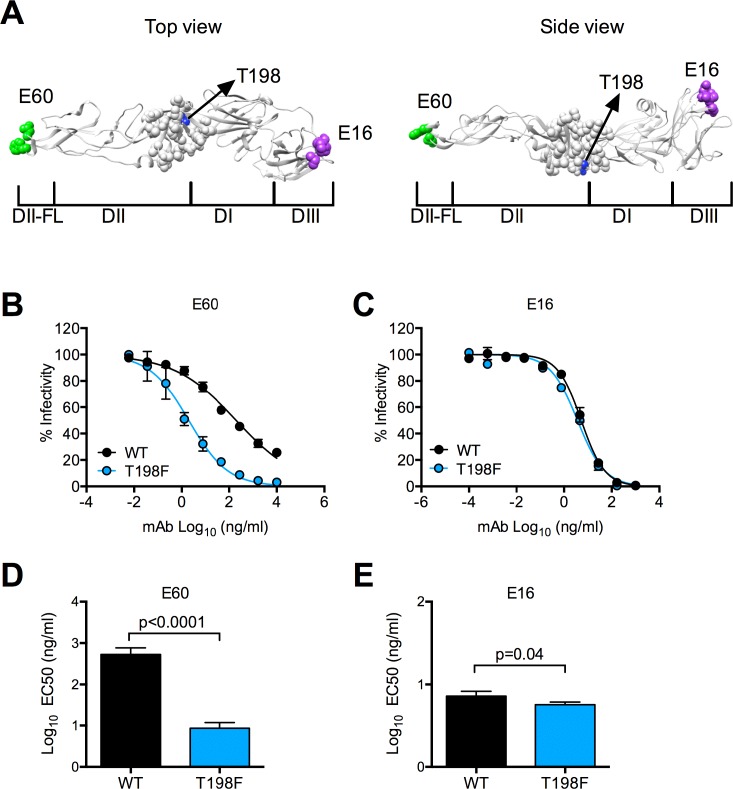
Neutralization sensitivity of WNV E T198F RVPs to mAbs. **(A)** Top (left panel) and side (right panel) views of the crystal structure of the WNV E protein monomer (PDB 2HG0) are shown, with domains I, II, and III (DI, DII, and DIII), and the fusion loop of DII (DII-FL) indicated below the structure. The side view was obtained by rotating the structure in the left panel 90 degrees towards the page. On the structure, amino acid residues important for recognition by mAb E60 in the DII-FL [58] and by mAb E16 [59] in DIII are indicated by the green and purple spheres, respectively. Gray spheres indicate residues within DI-DII hinge that were mutated for epitope mapping studies. The blue spheres and black arrow indicate the location of threonine at E protein residue 198 described in this study **(B)** Representative dose-response neutralization curves for WT and T198F WNV RVPs tested concurrently against mAbs E60 and **(C)** E16. The y- and x-axes indicate percent infectivity and mAb concentration, respectively. Infectivity was normalized to levels observed in the absence of antibody. Error bars indicate the range of infectivity from duplicate wells. We performed paired t-tests to compare the EC50 values of **(D)** E60 and **(E)** E16 against WT and T198F RVPs obtained from six independent experiments performed in duplicate. Error bars indicate the standard error of the mean (SEM).

To identify epitopes targeted by NAbs in flavivirus-immune sera, we created a library of WNV reporter virus particles (RVPs) [57] containing mutations at solvent-accessible E protein residues for use in neutralization studies. We focused on residues within the DI-DII hinge region, which has been shown to be an important target for many potently neutralizing antibodies against both WNV and DENV [23, 24, 33]. Because virion maturation state and conformational dynamics may modulate epitope accessibility and neutralization sensitivity indirectly, we performed a series of control experiments, as described previously [26], to identify pleiotropic effects of mutations on the overall antigenic structure of virions. These experiments identified a mutation at E residue 198 (T198F, **Fig 1A**) that unexpectedly modulated sensitivity to neutralization by the DII-fusion loop (DII-FL)-reactive mAb, E60, despite being located distally from the predicted epitope [58]. RVPs incorporating T198F were 68-fold more sensitive to E60 neutralization than were wild type (WT) RVPs (average EC50 = 13.1 and 881 ng/ml for T198F and WT RVPs, respectively, p< 0.0001, **Fig 1B and 1D**). In contrast, there was little difference in sensitivity to neutralization by mAb E16 (average EC50 = 5.9 and 8.1 ng/ml for T198F and WT RVPs, respectively, **Fig 1C and 1E**), which binds an accessible epitope on the DIII lateral ridge (**Fig 1A**) [59–62].

Neutralization studies with antibodies that bind poorly accessible epitopes such as those in DII-FL [46, 58, 63] often reveal incomplete neutralization even at saturating antibody concentrations [45]. As flavivirus neutralization occurs when the number of antibodies bound to the virion exceeds a stoichiometric threshold [62], incomplete neutralization may reflect structural heterogeneity among a genetically homogeneous RVP population that limits epitope accessibility. Virions that are not bound by antibodies at a stoichiometry sufficient for neutralization remain infectious [62]. We noted that a significantly smaller fraction of T198F RVPs remained infectious at the highest E60 concentration tested (10 μg/ml) compared to WT (5.5% and 26.7% for T198F and WT RVPs, respectively, p = 0.001, **Fig 1B**). These results demonstrate that the T198F mutation increases the accessibility of a cryptic epitope in DII-FL.

### Increased neutralization sensitivity of WNV T198F RVPs to mAb E60 is not explained by changes in virion maturation

**Fig 2:**
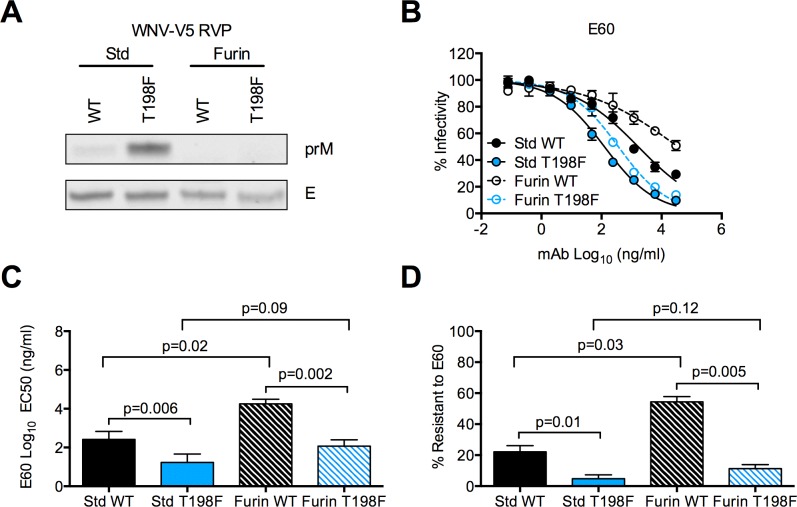
Effect of virion maturation state on neutralization sensitivity of WNV E T198F RVPs. **(A)** We assessed the level of V5-tagged prM in WNV RVPs prepared using standard (Std) conditions, or those increasing the efficiency of prM cleavage (Furin) by SDS-PAGE and Western blotting of pelleted virions using a mouse monoclonal antibody against V5 (top panel). The level of E protein (bottom panel), as detected by mouse monoclonal antibody, 4G2, was used as a loading control for each RVP preparation. Data are representative of three independent experiments performed with independent RVP preparations. **(B)** We concurrently tested Std and Furin preparations of WT and T198F RVPs for sensitivity to neutralization by mAb E60. Representative dose-response neutralization curves are shown, with the y- and x-axes representing percent infectivity and mAb concentration, respectively. Infectivity was normalized to levels observed in the absence of antibody. Error bars indicate the range of infectivity from duplicate infections. **(C)** Mean EC50 values for E60 against Std or Furin preparations of WT and T198F RVPs. Error bars represent the SEM. The indicated p-values were obtained from paired t-tests. **(D)** Mean percentages of Std or Furin WT and T198F RVPs resistant to neutralization at the highest concentration of E60 tested (10 μg/ml). Error bars represent the SEM. The indicated p-values were obtained from paired t-tests. For **(C)** and **(D)**, mean values were obtained from three independent experiments performed in duplicate using independent RVP preparations.

The neutralizing activities of many mAbs, including those targeting DII-FL, can be modulated by the efficiency of virion maturation [45, 46, 64]. Specifically, virions that retain uncleaved prM often are more sensitive to neutralization by antibodies that bind epitopes predicted to be poorly accessible on the mature virion. For example, increasing the efficiency of prM cleavage (**Fig 2A**) reduced the sensitivity of WT WNV RVPs to neutralization by E60 relative to a standard WT RVP preparation (~70-fold reduction in EC50, p = 0.02, **Fig 2B and 2C**), as detailed previously [26, 45, 47]. To investigate whether T198F modulates the efficiency of prM cleavage during virion maturation, we analyzed the prM content of RVPs produced using standard conditions (Std-RVP) and in the presence of over-expressed human furin (Furin-RVP). Although three independent preparations of Std T198F RVPs contained an average of five times the level of uncleaved prM compared to that of WT RVPs prepared in parallel, furin over-expression in RVP producing cells resulted in efficient prM cleavage for both WT and T198F RVPs (**Fig 2A**), suggesting that the increased prM content of Std T198F RVPs was not due to an inability of the virion to adopt conformations in which the prM cleavage site was accessible during egress. Moreover, increased prM content was not sufficient to explain the greater sensitivity of T198F RVPs to neutralization by E60, as Furin T198F RVPs were more sensitive to neutralization by E60 than were Furin WT RVPs (148-fold reduction in EC50, p = 0.002, **Fig 2B and 2C**), despite undetectable levels of uncleaved prM **(Fig 2A)**.

Consistent with prior studies [26, 45, 47], increasing the efficiency of prM cleavage increased the proportion of WT RVPs that were resistant to E60 neutralization at the highest mAb concentration tested (22% and 55% for Std and Furin WT RVPs, respectively, p = 0.03, **Fig 2B and 2D**). In contrast, we observed only a minimal difference between the proportion of Std and Furin T198F RVPs resistant to neutralization at saturating concentrations of E60 (5% and 11%, respectively, p = 0.12, **Fig 2B and 2D**). Additionally, in contrast to the 70-fold difference in EC50 between Std and Furin WT RVPs, there was a much smaller (7-fold) difference in EC50 between corresponding preparations of T198F RVPs (p = 0.09, **Fig 2B and 2C**). Altogether, these data suggest that the large increase in sensitivity of T198F RVPs to neutralization by E60 was not simply due to increased retention of uncleaved prM. Furthermore, the reduced impact of maturation state on E60 recognition of WNV T198F suggests that the E60 DII-FL epitope is distinctly displayed by this variant.

### Increased neutralization sensitivity of WNV T198F RVPs is not explained by a specific change in amino acid chemistry

**Fig 3:**
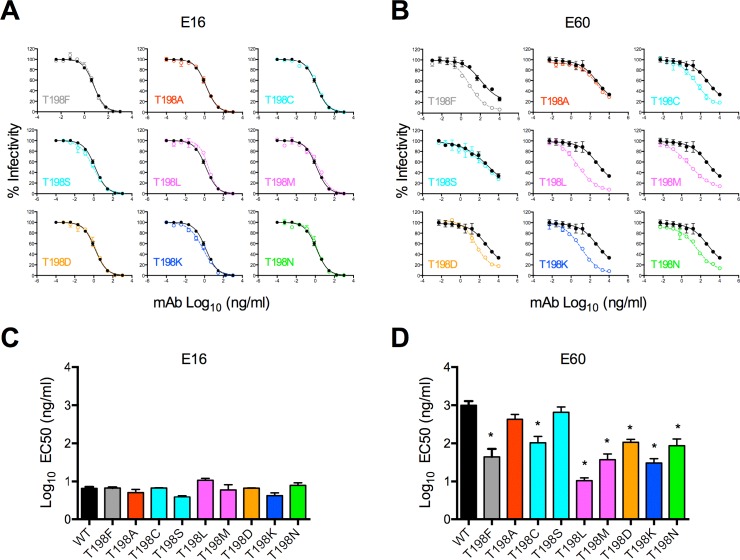
Effect of amino acid chemistry on neutralization sensitivity of WNV E T198 RVP variants. We tested WT WNV RVPs containing threonine at E residue 198 (black curves) for sensitivity to neutralization by mAbs **(A)** E16 and **(B)** E60 concurrently with RVPs incorporating amino acid variants representing distinct chemical groups at residue 198, including aromatic (F; grey), small (A; red), nucleophilic (C, S; cyan), hydrophobic (L, M; magenta), acidic (D; orange), basic (K; blue), and amide (N; green). Error bars indicate the range of infectivity from duplicate infections. Percent infectivity versus mAb concentration is shown in each graph. Infectivity was normalized to levels observed in the absence of antibody. We compared the mean EC50 values for **(C)** E16 and **(D)** E60 against each variant to those of WT RVPs using a one-way ANOVA followed by Dunnett’s multiple comparisons test. Data were obtained from three independent experiments performed in duplicate. Error bars indicate the SEM. The color scheme for distinct amino acid chemical groups corresponds to that in **(A)** and **(B)**. *, p<0.05.

We investigated whether the increased neutralization sensitivity of WNV T198F was dependent on amino acid chemistry at this position. In addition to T198F, we produced Std WNV RVPs in which the threonine at position 198 was replaced with amino acids containing small (A), nucleophilic (C, S), hydrophobic (L, M), acidic (D), basic (K), or amide (N) side chains. Each of these variants resulted in infectious RVPs, with titers within 2-fold of the WT control produced in parallel (p = 0.47, **S1 Fig**). Compared to WT, all T198 RVP variants were similarly sensitive to neutralization by mAb E16 (<2-fold difference in EC50, **Fig 3A and 3C**). However, when tested against E60, neutralization sensitivity varied among RVP variants, although there was no clear correlation with amino acid chemistry. For example, T198F, T198M, T198K resulted in a similar (~50-fold) reduction in EC50 despite incorporating amino acids with distinct side chain characteristics (**Fig 3B and 3D**). Importantly, every T198 variant except for T198A and T198S resulted in a significant increase in sensitivity to neutralization by E60 to varying extents (~10 to 100-fold reduction in EC50 compared to WT, **Fig 3B and 3D**). Thus, the increased sensitivity of T198F RVPs to neutralization by E60 was not linked to a particular change in amino acid chemistry at WNV E residue 198.

### Mutation of WNV E residue 198 reduces the infectious half-life of RVPs in solution

**Fig 4:**
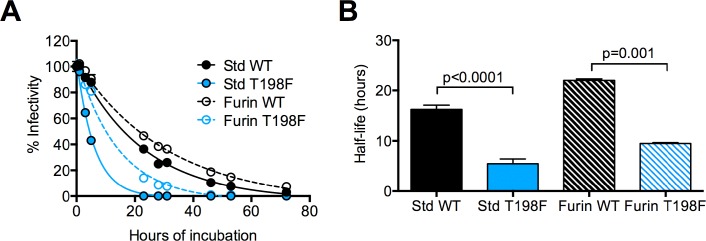
Stability of WNV E T198F RVPs. **(A)** Intrinsic decay of the infectivity of WT and T198F RVPs. Standard or Furin RVP preparations were equilibrated at 37°C for 1 h and further incubated for additional lengths of time as indicated on the x-axis, after which aliquots were harvested and frozen. Samples from each time point were thawed concurrently and used to infect Raji-DC-SIGN-R cells. Data were normalized to the infectivity of RVPs incubated at 37°C for 1 h and fitted to a one-phase exponential decay curve. Representative decay curves for WT and T198F RVPs are shown. Error bars indicate the SEM from triplicate infections. **(B)** Paired t-tests were used to compare the half-life of infectivity of WT and T198F RVPs. Shown are mean half-life values obtained from three independent experiments performed in triplicate. Error bars represent the SEM.

We hypothesized that the increased accessibility of the E60 DII-FL epitope on T198F virions was due to changes in virus conformational dynamics or ‘breathing,’ which allows the transient display of poorly exposed epitopes [47, 54]. A potential consequence of virus breathing is a reduction in virus stability that can be inferred by the loss of infectivity over time (or ‘intrinsic decay’) at physiological temperatures, as has been established for picornaviruses [65, 66]. Among the ensemble of conformations sampled by a virion at equilibrium, a subset may result in irreversible structural changes that are incompatible with infectivity for a given cell type. We therefore investigated whether the T198F mutation altered the functional stability of WNV RVPs. Following incubation at 37°C for up to 72 hours, the infectivity of WT and T198F RVPs collected periodically was determined by titrating viruses on Raji-DCSIGN-R cells (**Fig 4A**). In agreement with previous findings, virion maturation state modulated the rate of intrinsic decay [47, 67]: the infectivity of Std RVPs decayed more rapidly compared to that of Furin RVPs for both WT and T198F. For Std RVPs, the T198F substitution resulted in a ~3-fold reduction in half-life relative to WT (average half-life of 5 versus 16 hours, respectively, p<0.0001, **Fig 4B**). Similarly, the infectivity of Furin T198F RVPs decayed at a rate that was approximately twice as fast as Furin WT RVPs (average half-life of 10 versus 22 hours, respectively, p = 0.001). These findings support the hypothesis that the T198F mutation alters the ensemble of conformations sampled by virions at equilibrium.

### Mutation at the corresponding residue of DENV, but not ZIKV, E protein modulates neutralizing antibody sensitivity and virion functional stability

**Fig 5:**
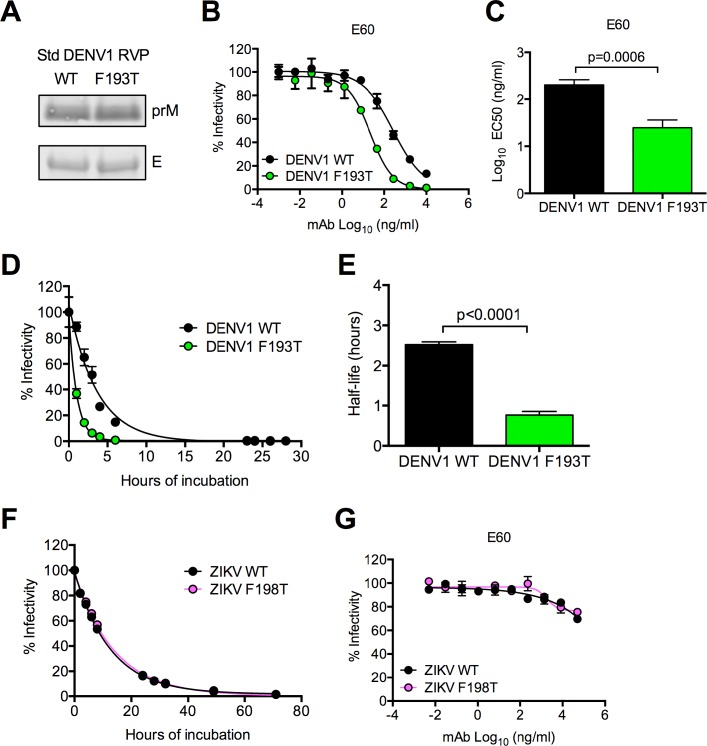
Characteristics of DENV1 E F193T and ZIKV E F198T RVPs. **(A)** We assessed the efficiency of prM cleavage of standard (Std) preparations of WT and F193T DENV1 RVPs by SDS-PAGE and Western blotting of pelleted virions using a mouse prM-reactive mAb (top panel). The level of E protein, as detected using mouse mAb 4G2, was used as a loading control (bottom panel). Data are representative of three independent experiments performed using independent RVP preparations. **(B)** Representative dose-response neutralization curves for Std DENV1 WT and F193T RVPs against mAb E60. Percent infectivity versus mAb concentration is shown. Error bars indicate the range of infectivity from duplicate infections. **(C)** Comparison of mean EC50 values of E60 against Std DENV1 WT and F193T RVPs using a paired t-test. Values were obtained from five independent experiments performed in duplicate. Error bars indicate the SEM. **(D)** Representative intrinsic decay curves of Std DENV1 WT and F193T RVPs. Experiments were performed as described in **Fig 4A**. Percent infectivity versus hours of incubation is shown. Error bars indicate the SEM obtained from triplicate infections of Raji-DC-SIGN-R cells. **(E)** Comparison of the mean half-life of infectivity of Std DENV1 WT and F193T RVPs using a paired t-test. Values were obtained from five independent experiments performed in triplicate. Error bars indicate the SEM. **(F)** Intrinsic decay of Std ZIKV WT and F198T RVPs. Experiments were performed as described in **Fig 4A**. Percent infectivity versus hours of incubation is shown. Decay curves shown are representative of three independent experiments. Error bars indicate the SEM obtained from triplicate infections of Raji-DC-SIGN-R cells. **(G)** Neutralization of Std ZIKV WT and F198T RVPs by mAb E60. Percent infectivity, normalized to levels observed in the absence of antibody, versus mAb concentration is shown. Curves shown are representative of four independent experiments. Error bars indicate the range of infectivity from duplicate infections.

The threonine at residue 198 in WNV and the phenylalanine found at the corresponding residue in DENV (193) and ZIKV (198) is highly conserved (99.9 to 100%) among 1989, 2692, and 104 sequences of WNV, DENV, and ZIKV naturally occurring isolates, respectively, available in the Virus Variation database [68]. We introduced the reciprocal mutation at this residue (F193T) into the Western Pacific strain of DENV1 to investigate whether it similarly affected antigenic structure and dynamics of the virion. The infectivity of standard RVP preparations of DENV1 F193T was reduced by ~10-fold as compared to WT DENVI (p = 0.001, **S1 Fig**). While prM cleavage was much less efficient for DENV than WNV, as reported previously [69, 70] **(Figs 2A and 5A**), the F193T mutation in DENV1 did not alter maturation efficiency; three independent standard preparations of WT and F193T DENV1 RVPs contained a similar level of prM (1.1-fold difference, **Fig 5A**). We next investigated the effect of F193T on the neutralization of DENV1 by E60 [58]. Similar to results with WNV T198F, DENV1 F193T RVPs were more sensitive to neutralization than WT RVPs (average EC50 = 25 ng/ml and 200 ng/ml for F193T and WT RVPs respectively, p = 0.0006, **Fig 5B and 5C**). Moreover, the F193T mutation resulted in a 3-fold reduction in the half-life of infectivity of DENV1 RVPs (average half-life of 2.5 and 0.8 hours for WT and F193T, respectively, p<0.0001, **Fig 5D and 5E**). In contrast to our findings with WNV and DENV1, mutation at the analogous residue (F198) of ZIKV E protein had no effect on sensitivity to neutralization by E60 or virion stability in solution (**Fig 5F and 5G)**. Together, these results suggest that a single residue in the DI-DII hinge of the E protein alters the exposure of a cryptic DII-FL epitope and the stability of flavivirus particles in solution in a context-dependent manner.

### Kinetic aspects of neutralization of WNV T198F RVPs

**Fig 6:**
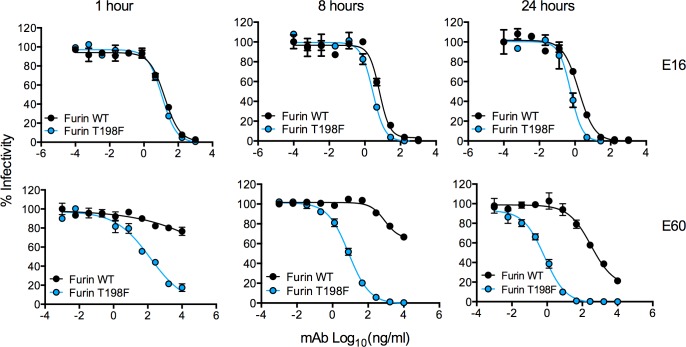
Kinetic aspects of neutralization of WNV E T198F RVPs. Furin WT and Furin T198F WNV RVPs were incubated with mAb E16 (top panel) or E60 (bottom panel) for 1 h at room temperature and were either immediately used to infect Raji-DC-SIGN-R cells or were further incubated at 37°C for additional lengths of time as indicated above the graphs prior to the addition of Raji-DC-SIGN-R cells. Percent infectivity versus mAb concentration is shown in each graph. Infectivity was normalized to levels observed in the absence of antibody. Error bars indicate the range of infectivity from duplicate infections. Neutralization curves shown are representative of five independent experiments performed in duplicate.

To explore further the possibility that the T198F mutation modulates WNV conformational dynamics, we performed kinetic neutralization assays, in which virus-antibody complexes were used to infect Raji-DC-SIGN-R cells immediately following a 1 h pre-incubation at room temperature, or further incubated at 37°C prior to addition of target cells (**Fig 6**). We studied only Furin RVP preparations containing little or no prM to eliminate the confounding effects of heterogeneity in virion maturation state on neutralization sensitivity (**Fig 2B)** [26, 45, 47]. As observed previously, increasing the virus-antibody incubation time for mAb E16, which binds an accessible epitope on DIII, resulted in only modest increases in neutralization potency against both WT and T198F RVPs [54]. In contrast, kinetic changes in neutralizing antibody potency were more pronounced with mAb E60. Increased sensitivity due to the T198F mutation was observed at all time points tested. For example, after 8 hours of incubation, we observed complete neutralization of Furin T198F RVPs, whereas a large proportion of Furin WT RVPs remained infectious at the highest E60 concentration tested. Even after 24 hours of incubation with E60, WT RVPs remained much less neutralized compared to T198F RVPs. These findings suggest that the T198F mutation alters accessibility of the E60 DII-FL epitope.

### T198F does not impair WNV replication *in vitro*

**Fig 7:**
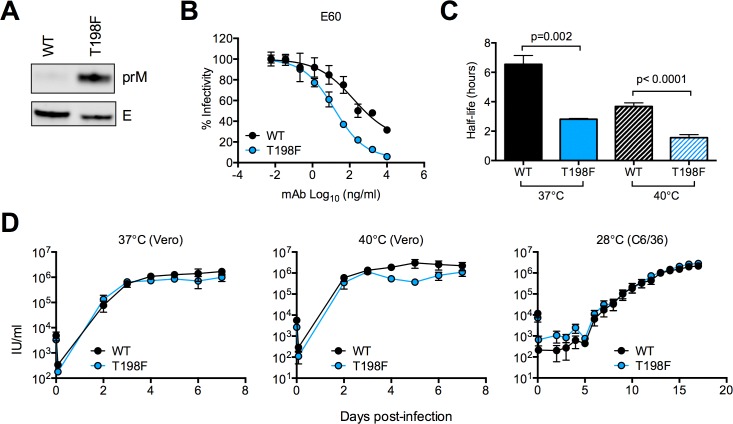
Characteristics of WNV E T198F fully infectious viruses. **(A)** The efficiency of prM cleavage of standard preparations of WT or T198F viruses was assessed by SDS-PAGE followed by Western blotting of pelleted virions, as described in **Fig 2**. **(B)** Sensitivity of standard preparations of WT and T198F WNV-GFP to neutralization by mAb E60. The y- and x-axes indicate percent infectivity and mAb concentration, respectively. Percent infectivity was normalized to levels obtained in the absence of antibody. Neutralization curves shown are representative of two independent experiments performed in duplicate using independent virus preparations. Error bars indicate the range of infectivity. **(C)** Paired t-tests were used to compare the half-life of infectivity of standard preparations of WT and T198F viruses following prolonged incubation at 37°C or 40°C as described in **Fig 4**. Shown are the mean half-life values obtained from three independent experiments performed in triplicate using independent virus preparations. Error bars indicate the SEM. **(D)** Standard preparations of WT and T198F viruses were used to infect Vero cells at 37°C or 40°C and C6/36 cells at 28°C at a MOI of 0.05. At the indicated time points, virus supernatant was collected and used to infect Raji-DC-SIGN-R cells to determine viral titers. Growth curves shown are based on average titers obtained from three independent experiments performed in duplicate using independent virus stocks. The y- and x-axes indicate virus infectious units (IU) per ml and days post-infection, respectively.

To extend our findings with RVPs capable of only a single round of infection, we investigated effect of the T198F mutation on standard preparations of fully infectious WNV encoding GFP [71]. As observed with RVPs (**Figs 1 and 2**), the T198F mutation reduced the efficiency of prM cleavage (**Fig 7A**), and resulted in increased sensitivity to neutralization by E60 (**Fig 7B**). Following prolonged incubation at 37°C, T198F reduced the infectious half-life of virions by ~2-fold relative to WT (average half-life of 2.8 and 6.5 hours, respectively, p = 0.002, **Fig 7C**), consistent with our observations with RVPs (**Fig 4**). As expected, raising the temperature at which viruses were incubated to 40°C reduced the infectious half-life of both WT and T198F RVPs (average half-life of 3.6 and 1.6 hours, respectively, **Fig 7C**) compared to incubation at 37°C; the 2-fold decrease in the half-life of T198F relative to WT viruses was maintained at 40°C (p<0.0001). Despite reduced stability in solution, T198F viruses nonetheless displayed similar growth kinetics as WT in Vero cells at 37°C and 40°C, and in mosquito (C6/36) cells at 28°C (**Fig 7D**), suggesting that the effect of T198F on virus stability in solution might be masked under conditions that allow efficient cell-cell spread of infection.

### *In vivo* effects of WNV T198F

**Fig 8:**
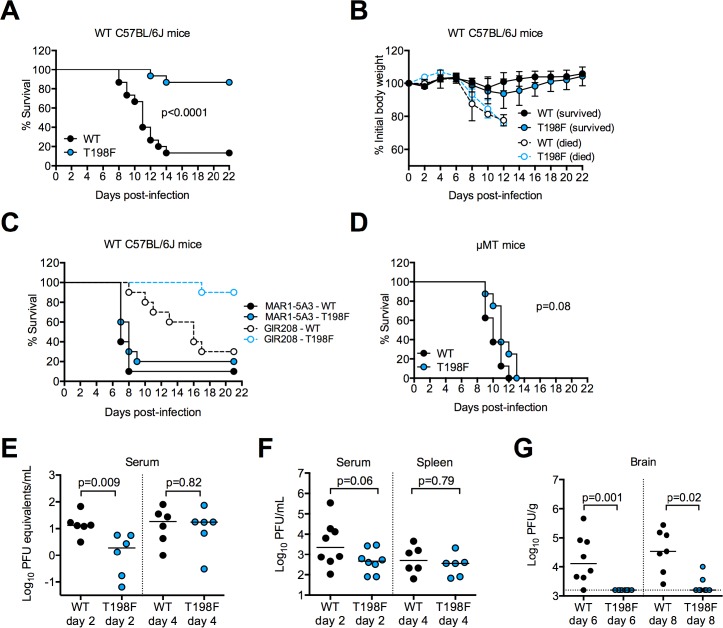
*In vivo* effects of WNV E T198F. Five-week old WT C57BL/6J mice were inoculated subcutaneously with 10^2^ FFU of WT (n = 15) or T198F (n = 15) WNV and monitored for **(A)** survival and **(B)** weight loss. Data are pooled from three independent experiments. Error bars in **(B)** represent the standard deviation. **(C)** Nine- to ten-week old WT C57BL/6J mice were injected via an intraperitoneal route with 0.5 mg each of blocking antibody against mouse IFN-α/β receptor (MAR1-5A3, n = 10) or an isotype control antibody against human IFN-γ receptor 1 (GIR-208, n = 10) one day prior to subcutaneous inoculation with 10^2^ FFU of WT or T198F WNV. Mice were monitored for survival up to 21 days post-infection. Data are pooled from two independent experiments. **(D)** Eight-week old μMT mice were inoculated subcutaneously with 10^2^ FFU of WT (n = 8) or T198F (n = 8) WNV and monitored for survival. Data are pooled from two independent experiments. **(E)** Five-week old WT C57BL/6J mice were infected with WT (n = 6) or T198F WNV (n = 6) as in **(A)**. Serum samples were collected on days 2 and 4 post-infection and viral burden was quantified by qRT-PCR. Horizontal lines across data points indicate the median viral burden. **(F)** Determination of infectious virus titer in the serum and spleen of five-week old WT C57BL/6J mice infected with WT (n = 8) or T198F (n = 8) virus at day 4 post-infection by plaque assay on Vero or BHK21 cells, respectively. Horizontal lines across data points indicate the median viral titer. **(G)** Five-week old WT C57BL/6J mice were infected with WT (n = 8) or T198F WNV (n = 8) as in **(A)**. Infectious titer in the brain at 6 and 8 days post-infection was determined by plaque assay on BHK21 cells. Horizontal lines across data points indicate the median viral titer. Data in **(E-G)** were pooled from two independent experiments. The p-values shown in **(A)** and **(D)** were obtained from a log-rank test; those in **(E-G)** were obtained by a Mann-Whitney test.

Because the T198F mutation did not impair WNV replication *in vitro*, we investigated its impact on pathogenesis in a well-established mouse model of infection [72]. We infected 5-week old C57BL/6J mice with either WT or T198F WNV and monitored survival for three weeks. In contrast to the high mortality rate (13 of 15) observed among mice infected with WT WNV, only 2 of 15 T198F-infected mice succumbed to infection (p<0.0001, **Fig 8A**). As expected, WT- and T198F-infected mice that died experienced rapid weight loss beginning at day 6 post-infection (**Fig 8B**). Weight loss was observed to a much lesser extent among mice that survived infection.

Type I interferon (IFN) and antibody responses have been shown to be critical for protection against lethal WNV infection [72–75]. We therefore investigated the outcome of T198F infection of mice treated with MAR1-5A3, a previously described blocking antibody against IFN-α/β receptor that prevents type I IFN-induced intracellular signaling *in vitro* and inhibits antiviral responses in mice [76, 77], and of congenic C57BL/6J mice that were genetically deficient in mature B cells and antibody (μMT strain). The attenuated phenotype of T198F in WT mice was not observed in MAR1-5A3-treated WT or μMT mice. Although T198F infection remained attenuated relative to WT infection (1/10 versus 7/10 deaths, respectively, p = 0.004, **Fig 8C**) in mice treated with GIR-208, an isotype control antibody targeting human IFNγ receptor, MAR1-5A3-treated mice were equally susceptible to lethal infection with WT or T198F virus (9/10 vs 8/10 deaths, respectively, p = 0.36, **Fig 8C**). Analogously, 8/8 μMT mice infected with either WT or T198F virus succumbed to lethal infection by day 12 and 13 post-infection, respectively (p = 0.08, **Fig 8D)**. Thus, the T198F mutation attenuates WNV in mice in a manner that is dependent on type I IFN signaling or B cell responses.

To investigate whether the T198F virus was attenuated due to rapid clearance, we collected serum samples at days 2 and 4 following infection of WT mice with either WT or T198F WNV. At day 2 post-infection, serum viral load was 7-fold lower in T198F- compared to WT-infected mice (p = 0.009, **Fig 8E**); a similar reduction in serum T198F infectious titer was observed at this time point (p = 0.06, **Fig 8F)**. However, by day 4 post-infection, T198F serum viremia reached WT levels (p = 0.82, **Fig 8E**). Moreover, there was no difference in the infectious titer of WT and T198F viruses harvested from spleens at 4 days post-infection (p = 0.79, **Fig 8F**). Despite similar levels of WT and T198F viremia by day 4 post-infection, viral burden in the brain of T198F-infected mice was severely reduced compared to WT-infected mice. By day 6, WT-infected mice had a median virus titer of 10^4^ PFU/g in the brain, whereas no infectious virus was detectable in the brain of T198F-infected mice (p = 0.001, **Fig 8G**). Infectious virus became detectable in the brain of 2 of 8 T198F-infected in mice by day 8, although at levels that were over 10-fold lower than those found in WT-infected mice on the same day (p = 0.02, **Fig 8G**). Sequence analyses of viruses isolated from the brain of T198F-infected mice revealed no reversion to WT. These results suggest that WNV containing the T198F mutation is suppressed early in infection and is attenuated for neuroinvasion or neurovirulence.

### Effect of natural antibodies on the infectious half-life of WNV in solution

**Fig 9:**
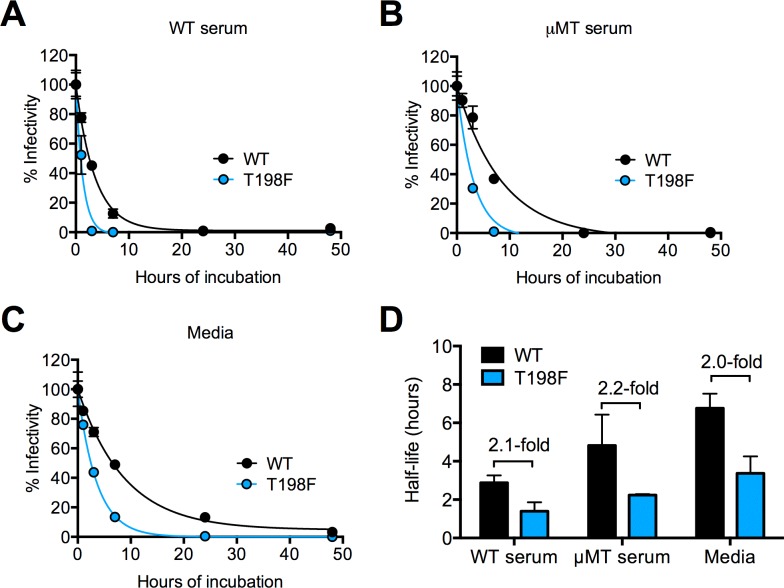
Effect of natural antibodies on WNV intrinsic decay. Representative intrinsic decay curves of fully infectious WT and T198F viruses in serum obtained from **(A)** naïve WT C57BL/6J mice, **(B)** naïve μMT C57BL/6J mice, and **(C)** media. Experiments were performed as described in **Fig 4A**. Error bars indicate the SEM from triplicate infections. **(D)** Average half-life values of WT and T198F viruses following prolonged incubation in serum obtained from naïve WT mice, naïve μMT mice, or media obtained from two independent experiments performed using independent serum samples and virus preparations. Error bars indicate the SEM. Fold-differences in half-life between WT and T198F WNV in each incubation condition are indicated.

As shown above, viremia in T198F-infected mice was reduced as early as day 2, but not by day 4 post-infection (**Fig 8E and 8F**). Because WNV-specific antibodies become detectable 4 days after infection [72, 73], we hypothesized that natural antibodies might accelerate the rate of T198F virus decay relative to WT. To test this hypothesis, we compared the stability of WT and T198F viruses incubated at 37°C in heat-inactivated serum samples obtained from naïve WT or μMT mice; viruses incubated in media were included as controls. We observed a ~2.3- and ~1.5-fold reduction in half-life of WT and T198F viruses in these serum samples, respectively, compared to incubation in media (**Fig 9**), suggesting that antibodies and other heat-resistant serum factors modulate the infectious half-life of WNV. Notably, the 2-fold reduction in half-life of T198F viruses compared to WT was observed following incubation in WT serum, μMT serum, and media (**Fig 9D**), suggesting that natural antibodies do not differentially modulate the rate of decay of WT and T198F WNV.

### Neutralizing activity of WNV-immune sera

**Fig 10:**
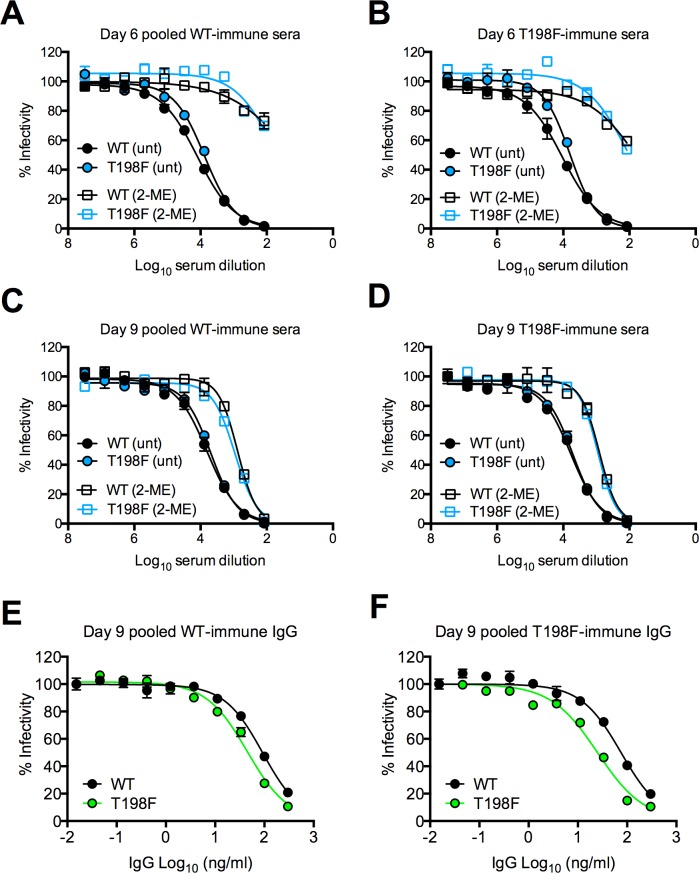
Neutralizing activity of WNV-immune sera. Sensitivity of WNV WT and T198F RVPs to neutralization by 2-mercaptoethanol (2-ME)-treated or untreated (unt) sera pooled from WT-immune (n = 5) **(A and C)** or T198F-immune (n = 5) **(B and D)** five-week old WT C57BL/6J mice. Sera were obtained at 6 **(A and B)** and 9 **(C and D)** days post-infection. IgG purified from pooled **(E)** WT-immune (n = 5) or **(F)** T198F-immune (n = 5) sera obtained from 9 days post-infection were tested for the ability to neutralize WNV WT and T198F RVPs. Error bars indicate the range of infection from duplicate wells. Data in **(A-F)** are representative of four independent experiments performed using independent RVP stocks.

To investigate whether neutralizing antibodies play a role in the attenuation of WNV T198F, we pooled serum samples from five WT- or T198F-infected WT mice for use in neutralization studies with WT and T198F RVPs. We compared serum samples obtained at days 6 and 9 after infection to distinguish neutralizing activity mediated by IgM and IgG, which become detectable at 4 and 8 days after infection, respectively [72, 73]. At both days 6 and 9, there were minimal differences (maximum of 1.2 fold change in EC50) in the ability of sera from WT- and T198F-infected mice to neutralize WT or T198F RVPs (**Fig 10A–10D**).

To distinguish IgM- versus IgG-mediated neutralizing activity, pooled serum samples from infected mice were either treated with 2-mercaptoethanol (2-ME), which preferentially degrades IgM [72, 78], or were used for IgG purification. As expected, treatment with 2-ME resulted in a large reduction (74–196 fold, **Fig 10A and 10B**) in the neutralization potency of serum samples obtained from WT- and T198F-infected mice at day 6, during which IgM, but not IgG is present [72, 73]. At day 9, when IgG is present, 2-ME treatment resulted in a smaller reduction in serum neutralization potency (4–8 fold, **Fig 10C and 10D**). As observed with untreated sera, at both days 6 and 9, there were minimal differences (maximum of 1.8 fold change in EC50) in the ability of WT- or T198F-immune sera treated with 2-ME to neutralize WT and T198F RVPs. Although T198F RVPs were slightly more sensitive (2–4 fold) to neutralization by IgG purified from day 9 sera compared to WT RVPs, this was observed for both WT- and T198F-immune IgG (**Fig 10E and 10F**), suggesting that infection with WNV T198F did not uniquely elicit NAbs that preferentially neutralized T198F viruses.

Finally, to directly study the impact of the T198F mutation on immunogenicity, we immunized WT C57BL/6J mice with WT or T198F RVPs capable of only a single round of infection. Pooled sera from WT or T198F RVP-immunized mice at either day 10 or 21 displayed limited differences in their ability to neutralize WT and T198F RVPs (**S2C–S2F Fig**). Similar results were observed with individual serum samples at day 21 post-immunization; although T198F was neutralized with a 3–4 fold greater potency compared to WT RVPs, this was observed for sera obtained from both WT and T198F RVP immunization groups (**S2G Fig**). These results suggest that neither infection nor immunization with T198F elicited unique NAb responses in mouse polyclonal sera.

## Discussion

Our study demonstrates that a single residue in the E protein DI-DII hinge regulates conformational dynamics in distinct flaviviruses, with relevant consequences *in vivo* for WNV infection and pathogenesis. Although conformational flexibility has been described for different virus families [53], the first evidence of the dynamic properties of flaviviruses came from structural studies of mAb 1A1D-2, which is capable of neutralizing multiple DENV serotypes, yet binds to an epitope in the β-strand of DIII that is not predicted to be fully accessible on the mature virus particle [79]. Monoclonal antibody 1A1D-2 can bind to DENV particles at 37°C, but not at 4°C, suggesting that at an elevated temperature, this mAb trapped a conformation on which the DIII β-strand epitope was otherwise not accessible at lower temperatures [79]. Consistent with the conformational flexibility of flaviviruses, subsequent studies showed that exposure of DENV2 virions to physiological temperatures in the absence of antibody results in the formation of an expanded ‘bumpy’ structure, on which E protein dimers are more loosely arranged and are rotated outwards relative to their orientation on fully mature particles [80, 81]. However, this ‘bumpy’ structure was not observed for all DENV strains or serotypes [81, 82], suggesting that sequence variation contributes to the structural pathways sampled by virus breathing at equilibrium.

The molecular determinants that govern flavivirus breathing have not been defined. We recently demonstrated that natural variation at residue 204 in DII of the DENV1 E protein explained large genotypic differences in sensitivity to neutralization by a mAb targeting a cryptic epitope in DIII [56]. In our current study, we identified a single mutation in the E protein DI-DII hinge of WNV (T198F) and DENV1 (F193T) that increased sensitivity to neutralization by mAb E60, which targets a poorly accessible epitope that includes the DII-FL. Because flavivirus neutralization occurs once the number of antibodies bound to the virion exceeds a stoichiometric threshold, neutralization potency depends not only on antibody affinity, but also on epitope accessibility [62]. Based on this model, antibodies targeting poorly exposed epitopes may not achieve complete neutralization even at saturating concentrations [45, 62], as observed for E60 against WT WNV. We observed that the T198F mutation markedly reduced the proportion of neutralization resistant WNV virions at high E60 concentrations. Compared to WT, increased accessibility of this epitope on T198F virions was less dependent on maturation state, which has been shown to indirectly modulate epitope accessibility [45, 46, 64]. Following prolonged incubation with E60 for up to 24 hours, WT WNV virions remained much less sensitive to neutralization than T198F virions. These results demonstrate that the E60 DII-FL epitope is displayed uniquely on T198F virus particles. Notably, increased sensitivity to neutralization by E60 also was observed by introducing a mutation at the corresponding residue of DENV1, but not ZIKV, suggesting that the molecular mechanisms governing conformational flexibility and/or FL exposure may be distinct for ZIKV, in agreement with recent neutralization studies with mAbs [31, 83–85]. The structural basis for these functional data cannot be inferred directly from our studies. The phenylalanine at position 193 and 198 of DENV and ZIKV, respectively play a space filling role (**S4 Fig**), while for WNV, the analogous threonine at this position projects outwards and is solvent exposed. Thus, the local structural environment likely contributes to the effects of amino acid substitutions at this position. Moreover, while the structures of mature ZIKV and DENV particles share many similarities, a distinguishing feature is an extended loop surrounding the glycan at ZIKV E residue 154, which has been hypothesized to limit accessibility of the adjacent DII-FL on the neighboring E protein [38].

Time-dependent increases in E60 neutralization potency were still apparent for T198F virions, suggesting that while this mutation increased the overall accessibility of a cryptic DII-FL epitope, it did not result in a grossly open ‘ground-state’ conformation. In support of this hypothesis, neutralization studies with an expanded panel of mAbs revealed that T198F (and DENV1 F193T) did not uniformly confer large increases in the potency of antibodies targeting distinct epitopes throughout the E protein (**S3 Fig**). Indeed, T198F resulted in a relatively modest increase in the neutralization sensitivity of WNV to mAb E53, which targets residues within the nearby DII bc-loop in addition to those within DII-FL [46, 58]. Together, these results suggest that T198F alters the ensemble of conformations sampled by WNV to increase the accessibility of poorly accessible epitopes within DII-FL. Consistent with alterations in conformational dynamics, T198F also impacted the functional stability of WNV virions. Although the molecular basis for the loss of virus infectivity (intrinsic decay) following prolonged incubation is not understood [47, 65, 66], we hypothesize this could be a consequence of virus breathing. Among the ensemble of conformations sampled by a dynamic virus, a subset may lead to irreversible changes in the E protein that impair viral infectivity. The more rapid intrinsic decay of T198F virions suggests that this mutation alters the conformational landscape in a manner that more frequently leads to irreversible changes in the E protein that are incompatible with infectivity.

Although studies of antibody reactivity and intrinsic decay have provided clues into the dynamic properties of flaviviruses, the consequences of virus breathing for viral replication and pathogenesis remain poorly understood. Our finding that the T198F mutation in WNV reduced the efficiency of prM cleavage from virions prepared under standard conditions suggests that virus breathing may affect the accessibility of the prM cleavage site during Golgi transit, thus contributing to the heterogeneity in the maturation state of released virus particles [41]. While the corresponding mutation in DENV1 (F193T) increased both sensitivity to E60 neutralization and the rate of intrinsic decay, prM cleavage efficiency in the context of DENV1 was unaffected. We previously demonstrated that the rate of intrinsic decay differs between WNV and DENV [47, 86], suggesting that sequence variation and the presence of uncleaved prM may alter the structural pathways sampled by flaviviruses. The possibility that virus breathing affects prM cleavage efficiency, perhaps by modulating access to the furin cleavage site on prM, further adds to the complex interplay among the determinants of flavivirus structural heterogeneity. Indeed, the reduced efficiency of prM cleavage of both WT and F193T DENV1 compared to WT WNV may reflect differences in the structural flexibility of DENV and WNV.

We demonstrated that altered virus breathing impacts pathogenesis. The T198F mutation attenuated WNV pathogenesis in WT mice, but not in mice treated with a monoclonal antibody targeting the IFN-α/β receptor or in congenic C57BL/6J mice deficient in B cells and antibody, suggesting that T198F attenuation is dependent on type I IFN- or B cell-mediated immunity. Our finding highlights the role of both innate and adaptive immune responses in protection against WNV lethal infection. Specifically, type I IFN signaling has been shown to be important in priming and enhancing B cell responses, in addition to its established role in innate antiviral defense [87–89]. Prior studies have demonstrated that both neutralizing and non-neutralizing WNV-specific antibodies can protect against lethal infection [72–74, 90]. For weakly neutralizing antibodies targeting DII-FL, protection is dependent on non-neutralizing mechanisms [90]. Our data indicate that T198F attenuation is not likely due to increased susceptibility to NAbs, suggesting a possible role for antibody effector functions.

T198F viremia was reduced as early as day 2 post-infection, before WNV-specific antibodies become detectable [72, 73], suggesting that this early viral suppression also might be due to differential effects of innate immune responses. Additionally, we previously found that the presence of even low concentrations of WNV-specific antibody can decrease the infectious half-life of virions *in vitro* [54], perhaps by trapping conformations that are incompatible with infectivity. Although we demonstrated that natural antibodies did not differentially affect the rate of intrinsic decay of WT and T198F viruses *in vitro*, it is possible that in the presence of other immune factors, even low concentrations of WNV-specific and/or natural antibodies may facilitate rapid viral clearance *in vivo* to limit dissemination to vital organs, as has been shown for other viruses [91]. Indeed, the suppression of T198F viremia very early in infection, though transient, was sufficient to limit CNS dissemination, which typically occurs between days 4 and 5 after infection [72, 74]. Finally, the stoichiometric requirements of prM cleavage for the production of infectious virus particles have not been defined. Although increased prM retention on T198F virus particles did not significantly impair infectivity *in vitro* (**Fig 7**), it is possible that decreased maturation efficiency of T198F may result in lower infectivity of key target cells *in vivo*, thus contributing to its attenuation.

Beyond impacting pathogenicity, the *in vivo* consequences of virus conformational dynamics are unexplored. The vector competence and extrinsic incubation period (time from infected blood meal to transmission) for both WNV [92–95] and DENV [96–99] are temperature-dependent, which could correspond to changes in the extent of virus breathing. It is intriguing to consider that the reduced infectious half-life of T198F WNV and F193T DENV1 virions in solution may result in less efficient transmission to mosquitoes during an infected blood meal, especially from a febrile animal, given that the rate of intrinsic decay is accelerated at elevated temperatures (**Fig 7C** and [86]). Indeed, sequence analyses reveal that WNV E residue 198 and the analogous DENV E residue 193 are highly conserved in nature [68]. The impact of changes in conformational dynamics on virus attachment to target cells also is unexplored. In addition to increased susceptibility to immune clearance, changes in conformational flexibility also might impair T198F virus interaction with host attachment factors in the central nervous system or in the vessels lining the blood-brain barrier [100].

T198F was neutralized slightly more potently than WT RVPs by both WT- and T198F-immune mouse sera **(Fig 10** and **S2 Fig**), suggesting that infection or vaccination with T198F did not skew the NAb response to preferentially neutralize T198F. Thus, although the T198F mutation impacts antigenicity as measured by changes in accessibility of a cryptic DII-FL epitope, its effects on immunogenicity are unclear. These results suggest, however, that antibodies targeting DII-FL do not significantly contribute to the overall neutralizing activity of polyclonal sera in mice. As the specificity of the polyclonal antibody repertoire elicited by flavivirus infection likely differs between mice and humans [22, 101–103], how changes in E protein conformational flexibility alter immunogenicity in humans remains to be determined. Recently, a number of potently neutralizing human monoclonal antibodies that target quaternary epitopes within or across flavivirus E protein dimers have been identified following natural infection or vaccination [23, 24, 27, 30, 31, 33, 34]. We speculate that the dynamic properties of E proteins have the potential to impact the exposure of these epitopes, and thus the induction of antibodies against them. The conformational flexibility of envelope proteins has been shown to modulate antibody recognition of HIV ([104]; among the different structures sampled by HIV envelope trimers at equilibrium, broad and potent NAbs preferentially target the highly ordered, ‘closed’ trimer conformation [104–106]. Based on these observations, current HIV immunogen design strategies to elicit broad and potent NAbs are focused on stably presenting the closed form of native envelope trimers [106–108]. Whether limiting conformational flexibility is a suitable strategy for flavivirus vaccine design awaits further studies.

Our ongoing studies aim to identify additional residues throughout the E protein that regulate conformational flexibility to facilitate studies on the impact of flavivirus breathing on immunogenicity and other aspects of flavivirus biology, including maturation, replication, and the pH threshold of fusion [109, 110]. We hypothesize that residues at the E protein hinge regions and dimer interface play critical roles in regulating virus breathing by virtue of their conformational flexibility [17, 18] and potential interactions that contribute to overall virion stability [111], respectively. The existence of antiviral compounds that inhibit virus breathing of selected picornaviruses suggests an important role for structural flexibility in the lifecycle of viruses [112–117]. Structural flexibility contributes to heterogeneity in the antigenic structure of virions by governing the exposure of cryptic epitopes that may be immunodominant [47, 54]. For example, antibodies that bind the conserved DII-FL are cross-reactive, poorly neutralizing antibodies with the potential to contribute to antibody dependent enhancement at high concentrations, which is especially relevant in the context of DENV infection [52, 118]. We have shown here for WNV and elsewhere for DENV [56] that exposure of cryptic epitopes can be modulated by amino acid substitutions at a distance. Thus, an improved understanding of the molecular determinants that regulate flavivirus breathing and the consequences of conformational dynamics on flavivirus biology has the potential to inform both the design of novel vaccines and identification of antiviral compounds.

## Methods

### Cell culture

HEK-293T (ATCC) and Vero (ATCC) cells were maintained in Dulbecco’s Modified Eagle medium (DMEM) containing 25 mM HEPES (Invitrogen) supplemented with 7% fetal bovine serum (FBS; Invitrogen) and 100 U/ml penicillin-streptomycin (P/S; Invitrogen). C6/36 (ATCC) cells were similarly cultured, except with the addition of 1X non-essential amino acids (Invitrogen). Raji-DC-SIGN-R cells (Raji B lymphoblast [ATCC] engineered to stably express DC-SIGN-R, Pierson lab [45, 54, 62, 119]) were cultured in RPMI 1640 medium containing Glutamax (Invitrogen) supplemented with 7% FBS and 100 U/ml P/S. HEK-293T, Vero, and Raji-DC-SIGN-R cells were maintained at 37°C in the presence of 7% CO_2_. C6/36 cells were maintained at 28°C in the presence of 7% CO_2_.

### Generation of E variants

We used a previously described expression vector encoding the structural genes (C-prM-E) of the WNV NY99 strain [57] as a template for mutagenesis. Initially, threonine at residue 198 of the WNV E protein was replaced with phenylalanine by site-directed mutagenesis using the *Pfu* Ultra DNA polymerase system (Agilent Technologies). The reciprocal mutation (F193T) was introduced into a previously described expression vector encoding the structural genes (C-prM-E) of the DENV1 Western Pacific strain [86]. Mutation at the analogous residue of the ZIKV E protein (F198T) was introduced into a plasmid encoding the structural genes of the ZIKV strain H/PF/2013 [120]. This plasmid is described elsewhere [121]. Additional amino acid variants were introduced at position 198 of the WNV E protein using primers containing a degenerate codon (NNN). PCR cycling parameters were as follows: 1 cycle of 95°C for 1 min; 18 cycles of 95°C for 50 s, 60°C for 50 s, and 68°C for 9 min; and 1 cycle of 68°C for 7 min. PCR products were treated with DpnI (New England BioLabs) for 3 h at 37°C, prior to transformation into Stbl2 cells (Invitrogen) and propagation at 30°C. The entire C-prM-E region of each construct was sequenced to ensure that no additional mutations were present.

### Production of RVPs

RVPs were produced by complementation of a GFP-expressing WNV sub-genomic replicon with plasmids encoding the structural genes of WNV, DENV, or ZIKV, as described previously [121, 122], with slight modifications. Briefly, HEK-293T cells were pre-plated in a low-glucose (1 g/liter) formulation of DMEM containing 25 mM HEPES (Invitrogen), 7% FBS, and 100 U/ml P/S, transfected with plasmids encoding the replicon and structural genes at a 1:3 ratio by mass using Lipofectamine 3000 (Invitrogen), and incubated at 37°C. For each microgram of DNA, 2 μl of Lipofectamine 3000 was used. Four hours post-transfection, cells were transferred to 30°C. Supernatant was harvested at 72–96 h post-transfection, passed through a 0.22 μm filter (Millipore), and stored at –80°C. To produce mature preparations of WNV RVPs containing low to undetectable prM, RVPs were produced as above by co-transfecting plasmids encoding the replicon, structural genes, and human furin at a 1:3:1 ratio. To detect prM in RVP preparations, a modified structural gene construct that encodes prM and E, with a V5 tag immediately downstream of the prM signal cleavage site [123] was used to complement a plasmid encoding capsid [57]. For immunization studies, 180 ml of transfection supernatant containing WT or T198F RVPs was passed through a 0.22 μm filter, layered on 20% sucrose (pH 7.4), and pelleted by ultracentrifugation at 32,000 rpm at 4°C for 5 h. The virus pellet was resuspended in 0.5 ml of PBS containing 1% BSA.

### Production of fully infectious WNV

Infectious WNV encoding a GFP reporter gene was produced using a previously described molecular clone system in which a DNA fragment encoding WNV structural genes is ligated into a GFP-expressing WNV replicon plasmid (pWNV-GFP-backbone V3) and transfected directly into HEK-293T cells [71]. Briefly, 1 μg each of the backbone and structural gene plasmids was digested with BamHI and BssHII, and ligated with T4 DNA ligase (New England Biolabs) in a final volume of 40 μl at 16°C overnight. Next, the entire unpurified ligation mixture was transfected directly into HEK-293T cells using Lipofectamine 3000 (Invitrogen). Cells were incubated at 37°C in the presence of 7% CO_2_. Viral supernatant was harvested at 48 and 72 h post-transfection, filtered using a 0.22 μm filter (Millipore), and stored at –80°C. To detect prM in fully infectious virus preparations, a DNA fragment encoding WNV structural genes was modified to express a V5 tag immediately downstream of the prM signal cleavage site and was used for virus production as described above.

### Determination of virus titer

Clarified virus-containing supernatant was serially diluted 2-fold in a total volume of 100 μl and used to infect 5 x 10^4^ Raji-DC-SIGN-R cells in an equal volume at 37°C. Cells were fixed in 1.8% paraformaldehyde at 48 h or 16 h following infection by RVPs or fully infectious viruses, respectively, and GFP-positive cells enumerated using flow cytometry. Virus titer was calculated using the linear portion of the virus-dose infectivity curve using the following formula: Infectious units (IU)/sample volume = (% GFP-positive cells) x (number of cells) x (dilution factor).

### Measuring growth kinetics of WNV

Viruses produced in HEK-293T cells using WNV-GFP-backbone V3 [71] as described above were used to inoculate Vero or C6/36 cells at a multiplicity of infection (MOI) of 0.05 for 2 h at the indicated temperatures, after which supernatant was collected to confirm the input virus titer. After washing twice with PBS to remove unbound virus, cells were further incubated at 37°C (Vero), 40°C (Vero), or 28°C (C6/36). At the indicated time points, virus supernatant was collected and clarified by centrifugation at 2000 rpm for 5 min. Virus titers were determined on Raji-DC-SIGN-R as described above.

### Neutralization assays

RVP or fully infectious virus stocks were diluted to a level of infectivity that ensures antibody excess (~5 to 10%) and incubated with serial dilutions of mAbs or heat-inactivated (56°C for 1 h) sera for 1 h at room temperature before addition of Raji-DC-SIGN-R cells. To investigate the kinetics of neutralization, virus-antibody complexes were further incubated for additional lengths of time at 37°C as indicated prior to addition of Raji-DC-SIGN-R cells. All infections were performed in duplicate at 37°C. At 48 h (RVP) or 16 h (fully infectious virus) post-infection, infectivity was scored as a percentage of GFP-positive cells by flow cytometry. Antibody dose-response curves were analyzed using non-linear regression with a variable slope (GraphPad Prism v 6.0g, GraphPad Software Inc.) to calculate the concentration of antibody (EC50) required to inhibit infection by 50%, or the maximum inhibition of infectivity achieved at the highest antibody concentration tested (‘% Resistant’).

### IgM- versus IgG-mediated neutralization

Serum samples were depleted of IgM by treatment with 0.1 M of 2-mercaptoethanol in 1X PBS for 1 h at 37°C, as described previously [72, 78]. Total IgG was purified from 50 μl sera pooled from WT-immune (n = 5) or T198F-immune (n = 5) five-week old WT C57BL/6J mice at day 9 post-infection using the Melon IgG purification kit (Thermo Scientific) in a final volume of 500 μl (1:10 dilution). Purified total IgG was quantified using a human IgG ELISA kit (Immunology Consultants Laboratory) for use in neutralization assays as described above.

### Intrinsic decay of infectivity

Viruses were diluted to a similar level of infectivity as used in neutralization assays, allowed to equilibrate at the indicated temperature for 1 h (reference) and sampled periodically for the next 48–72 h. At each time point, aliquots were collected and stored at –80°C. All frozen samples were thawed simultaneously and used to infect Raji-DC-SIGN-R in triplicate to assess infectivity as described above. Infection was normalized to the level observed at the initial reference time point and fitted with a one-phase exponential decay curve (GraphPad Prism v 6.0g, GraphPad Software Inc.) to estimate the infectious half-life.

### Analysis of RVP maturation state

The level of prM in RVP preparations was determined by SDS-PAGE and Western blot analysis, as previously described [64, 123]. Briefly, RVPs were concentrated and partially purified by ultracentrifugation at 4°C (32,000 rpm for 5 h) through a 20% sucrose cushion, followed by re-suspension in TNE buffer (50 mM Tris, 140 mM NaCl, 5 mM EDTA, pH adjusted to 7.4) containing 1% Triton-X100. WNV and DENV1 E proteins were detected by a cross-reactive DII-FL reactive mouse monoclonal antibody, 4G2 (1 μg/ml). WNV prM-V5 was detected using a 1:5000 dilution of a mouse monoclonal antibody targeting V5 (Invitrogen), while DENV1 prM was detected using mouse monoclonal antibody, prM22 (0.5 μg/ml) [124]. IRDye 800CW goat-anti mouse IgG (LI-COR Biosciences) diluted 1:2500 was used as a secondary antibody. Protein bands were visualized and quantified using the Odyssey infrared imaging system (LI-COR Biosciences).

### Mouse experiments

C57BL/6J mice were purchased from Jackson Laboratories (Bar Harbor, ME) and congenic μMT B cell-deficient were bred at Washington University under pathogen-free conditions. Five-week old WT C57BL/6J mice or eight-week old μMT mice were inoculated subcutaneously via footpad injection with 10^2^ focus-forming units (FFU) of WNV NY99 WT or T198F, and monitored daily for survival. Where indicated, C57BL/6J mice were injected via an intraperitoneal route with 0.5 mg of a mouse monoclonal antibody targeting mouse IFN-α/β receptor (MAR1-5A3) [76] or an isotype control mouse antibody targeting human IFN-γ receptor 1 (GIR-208) one day prior to infection. Purified LPS-free monoclonal antibodies MAR1-5A3 and GIR-208 were purchased from Leinco Technologies. WT and T198F viral stocks were generated by *in vitro* transcription of an infectious two-plasmid cDNA clone as previously described [125]. The T198F mutation was introduced into plasmid pWN-AB, which consists of the 5'-UTR and structural genes, by site-directed mutagenesis as described above. For immunization studies, five-week old C57BL/6J mice were injected via an intraperitoneal route with 50 μl of WNV WT or T198F RVPs normalized by infectivity and relative E protein content as determined by antigen capture ELISA. Serum from immunized mice collected at days 10 and 21 were analyzed in neutralization studies.

### Antigen capture ELISA for WNV E protein

High-binding 96-well plates (Corning) were coated with 3 μg/ml humanized mAb E16 in 100 μl coating buffer (100 mM BupH Carbonate Bicarbonate Buffer, Fisher) with pH adjusted to 9.6. Plates were washed six times with PBS containing 0.05% Tween 20 followed by incubation with 100 μl blocking buffer (PBS, 3% non-fat dry milk, and 0.05% Tween 20). RVPs were serially diluted 2-fold starting at a 1:100 dilution in 100 μl blocking buffer, added to plates, and incubated for 1 h at 37°C. Plates were washed again and were incubated with 100 μl of mouse mAb E16 diluted in blocking buffer (2 μg/ml) for 1 h at 37°C. Following washing, 100 μl of HRP-conjugated goat anti-mouse IgG (Thermo Scientific) diluted 1:10,000 in blocking buffer were added to plates and incubated for 1 h at 37°C. One-step Ultra TMB-ELISA (Thermo Scientific) substrate was added (100 μl/well) and incubated for six minutes at room temperature in the dark. The reaction was stopped by the addition of 100 μl 1N hydrocholoric acid (Fisher) and read on a plate reader (BioTek Synergy H1) at a wavelength of 450 nm.

### Measurement of viral burden

On the indicated day post-infection, mice were sacrificed and organs collected following extensive perfusion with PBS. Organs were weighed, homogenized using a bead-beater apparatus, and titrated by plaque assay on BHK-21 cells [126]. Viral burden in serum samples was measured by plaque assay on Vero cells, and viral RNA from serum was isolated using the Viral RNA Mini Kit (Qiagen) and measured by quantitative fluorogenic reverse-transcription PCR as described previously [126].

### Statistical analysis

Statistical analyses were performed using GraphPad Prism v 6.0g (GraphPad Software Inc.). For results of *in vitro* experiments, paired t-tests or a one-way ANOVA followed by Dunnett’s multiple comparisons test was used, for two or more comparisons, respectively. For survival analysis, Kaplan-Meier curves were plotted and analyzed by the log rank test. Mouse serum, spleen, and brain viral loads and titers were compared using the Mann-Whitney test.

### Ethics statement

Experiments were approved and performed in accordance with the recommendations in the Guide for the Care and Use of Laboratory Animals of the National Institutes of Health. The protocols were approved by the Institutional Animal Care and Use Committee at the Washington University School of Medicine (Assurance number A3381-01).

## Supporting information

S1 Fig Infectivity of WNV T198 and DENV1 F193 RVP variants. **(A)** Threonine at WNV E residue 198 was replaced with various amino acids representing distinct chemical groups, including aromatic (F; gray), small (A; red), nucleophilic (C, S; cyan), hydrophobic (L, M; magenta), acidic (D; orange), basic (K; blue), and amide (N; green). These variants were used to create RVPs and infectivity was determined concurrently with WT WNV on Raji-DC-SIGN-R cells. Values shown are mean titers obtained from 4–13 independent RVP preparations. Titers are expressed as infectious units per ml (IU/ml) as determined by the formula described in the methods section. Error bars indicate the SEM. The p-value shown was obtained from a one-way ANOVA. **(B)** Infectivity of DENV1 F193T was determined concurrently with WT DENV1 as described in **(A)**. Values shown are mean titers obtained from five independent RVP preparations. Error bars indicate the SEM. The p-value shown was obtained from a paired t-test.
(TIFF)

S2 Fig Neutralizing activity of sera from mice immunized with WNV WT or T198F RVPs. Following concentration and partial purification through a sucrose cushion by ultracentrifugation, WT and T198F RVPs were analyzed for **(A)** infectivity on Raji-DCSIGN-R cells and (**B**) E protein content by antigen capture ELISA. Data are representative of two independent experiments performed with independent RVP preparations. Error bars in panels A and B represent the range of titers in infectious units/ml (IU/ml) and of OD 450 values from duplicate wells, respectively. Pooled serum samples collected from five-week old WT C57BL/6J mice at days 10 **(C, D)** and 21 **(E, F)** following immunization with either **(C, E)** WT (n = 10) or **(D, F)** T198F (n = 10) RVPs normalized by infectivity and E protein content were tested for neutralizing activity against WT and T198F RVPs. Shown are representative dose-response neutralization curves. Error bars indicate the range of infection of duplicate wells. Data are representative of three independent experiments. **(G)** Individual serum samples obtained from day 21 post-immunization with WT or T198F RVPs (n = 10 each) were tested for neutralizing activity against WT or T198F RVPs. Data points represent reciprocal serum dilutions required to inhibit infectivity by 50% (NT50) for each sample. Solid horizontal bars represent the geometric mean and 95% confidence intervals. The dotted horizontal line represents the lowest serum dilution tested. P-values shown were obtained from paired t-tests.
(TIFF)

S3 Fig Sensitivity of WNV T198F and DENV1 F193T RVPs to neutralization by an extended panel of mAbs. Standard preparations of **(A)** WNV or **(B)** DENV1 WT and mutant RVPs were concurrently tested for neutralization sensitivity against a panel of mAbs targeting distinct epitopes as indicated in the first two columns. The next two columns indicate the mean and SEM of EC50 values for WT and mutant RVPs, respectively, obtained from 3–10 independent experiments performed in duplicate, followed by p-values from paired t-tests. The final column indicates the fold-change in EC50 of each mAb against mutant relative to WT RVPs. Values greater than one indicate increased neutralization sensitivity of mutant relative to WT RVPs. The fold change in EC50 values is color-coded as indicated in the key in panel A.
(TIFF)

S4 Fig **(A)** Top (upper panel) and side (lower panel) views of the E protein dimer of DENV (PDB 1OAN) with residue F193 indicated by black spheres. The side view was obtained by rotating the dimer in the upper panel 90 degrees towards the page. Domains I, II, and III are indicated in red, yellow, and blue, respectively, with the fusion loop of DII shown in green. **(B)** Superimposition of E residues F193 of DENV (magenta), F198 of ZIKV (cyan, PDB 5JHM), and T198 of WNV (orange, PDB 2HG0). Residues F193 and F198 of DENV and ZIKV, respectively point away from the viral membrane towards the neighboring E protein within the dimer, while T198 of WNV projects outwards and is oriented parallel to the membrane surface.
(TIFF)
